# Supplementary material for: Enhanced Biosafety of the Sleeping Beauty Transposon System by Using mRNA as Source of Transposase to Efficiently and Stably Transfect Retinal Pigment Epithelial Cells
Source: Biomolecules. 2023 Apr 7;13(4):658. doi: 10.3390/biom13040658 (PMC10135490; doi:10.3390/biom13040658)
Supplement: Supplementary file 1 [file biomolecules-13-00658-s001.zip › biomolecules-2181715-supplementary.pdf]

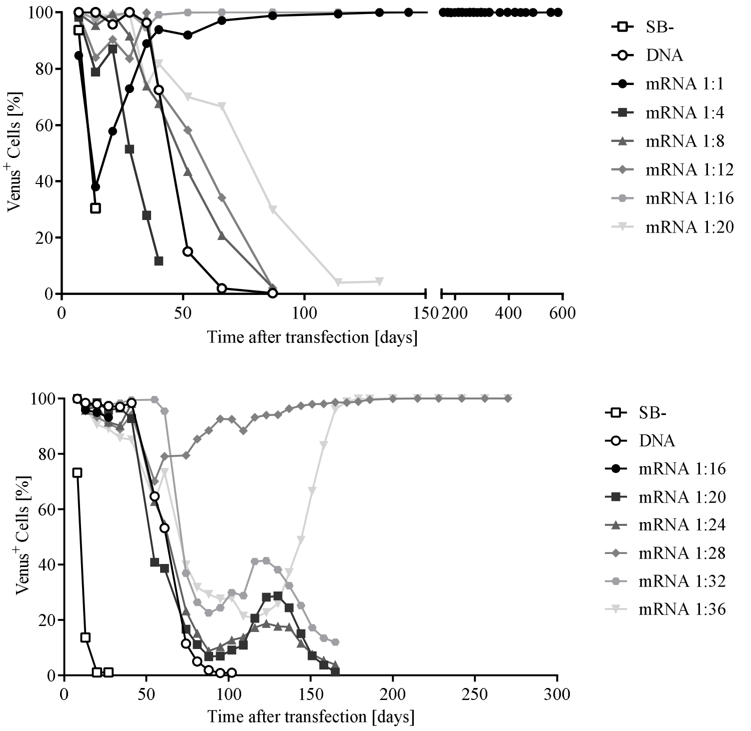

Figure S1: Analysis of transfection efficiency and long-term expression in ARPE-19 cells transfected with pT2-CAGGS-Venus. Complete data sets of the fluorescence analysis, shown in figure 2, of all samples over the whole period of the experiments (first experiment at the top, second experiment at the bottom). Cultures were terminated when the percentage of fluorescent cells decreased under 10%.

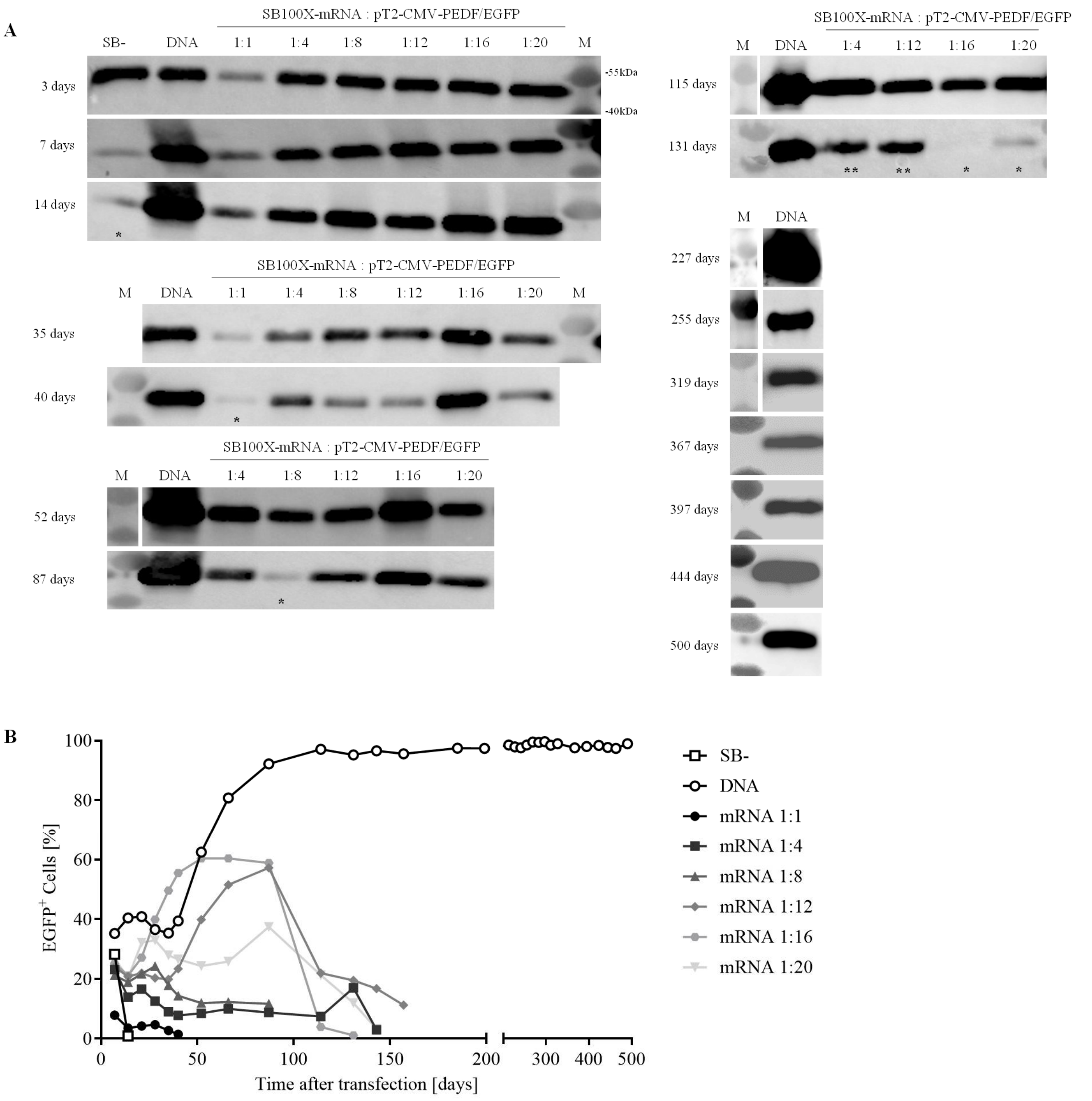

Figure S2: Analysis of transfection efficiency and long-term expression in ARPE-19 cells transfected with pT2-CMV-PEDF/EGFP. Complete data sets of the results shown in figure 4, first experiment. (A) Western Blot images of all analyzed timepoints. (B) Fluorescence analysis of all samples over the whole period of the experiment. Cultures were terminated when only low or no rPEDF secretion was detectable by Western Blot (labeled with *) or when the percentage of fluorescent cells decreased under 10% (labeled with **).

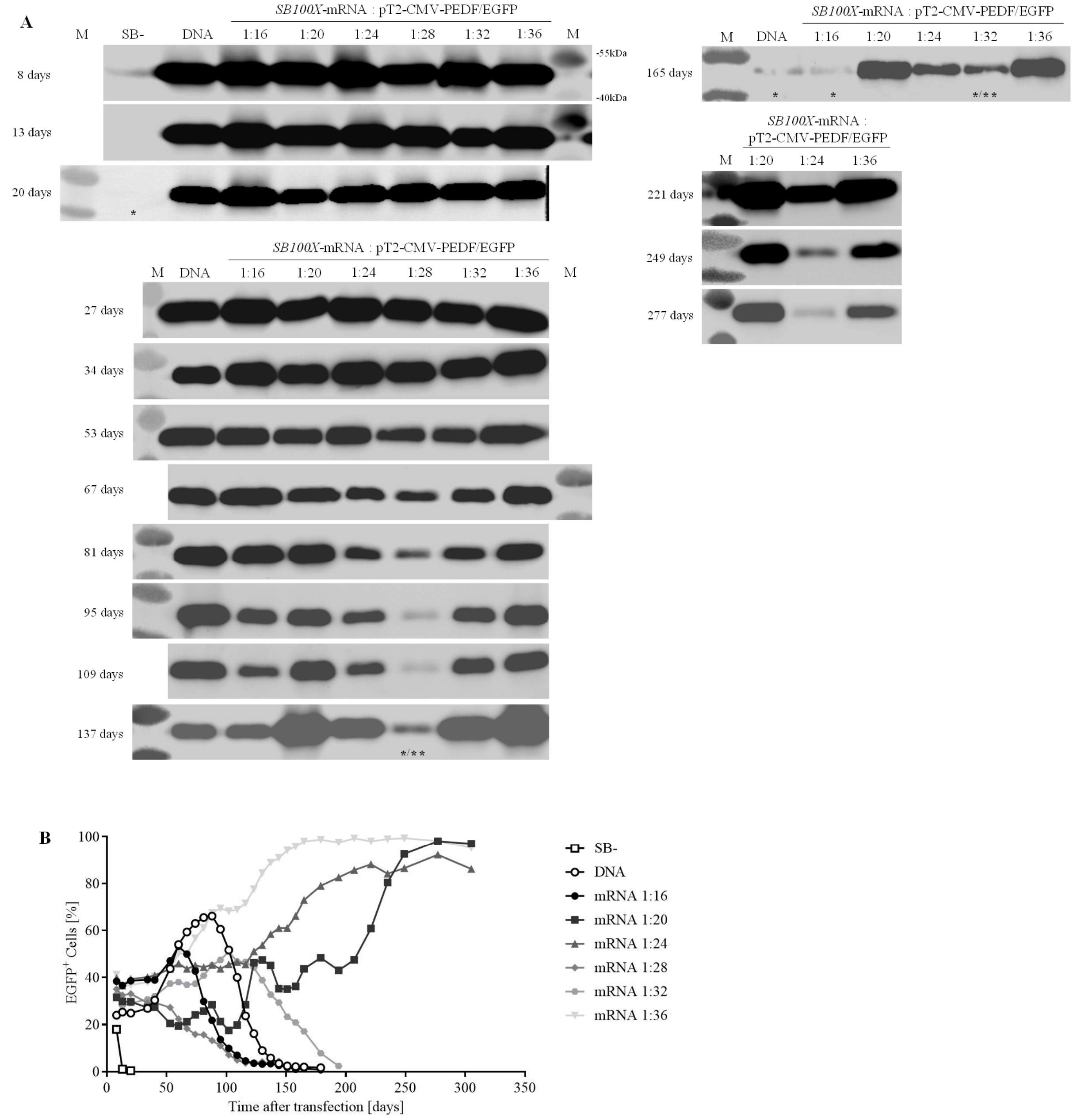

Figure S3: Analysis of transfection efficiency and long-term expression in ARPE-19 cells transfected with pT2-CMV-PEDF/EGFP. Complete data sets of the results shown in figure 4, second experiment. (A) Western Blot images of all analyzed timepoints. (B) Fluorescence analysis of all samples over the whole period of the experiment. Cultures were terminated when only low or no rPEDF secretion was detectable by Western Blot (labeled with *) or when the percentage of fluorescent cells decreased under 10% (labeled with **).

Table S1 : Donor characteristics of human RPE cells used for transfection with SB100X-mRNA and pT2-CAGGS-Venus or pT2-CMV-PEDF/EGFP.

Donor No.	Transfected with	Age [years]	Gender	Post-mortem time [hours]	Cultivation time before transfection [days]
**1**	Venus, PEDF	75	male	67.2	19
**2**	Venus, PEDF	54	female	67.8	19
**3**	Venus, PEDF	67	male	31.2	13
**4**	Venus, PEDF	66	female	22.7	20
**5**	Venus	74	male	38.1	41
**6**	Venus	73	male	13.1	36
**7**	PEDF	65	female	39.3	39
**8**	PEDF	60	female	47.9	14
**9**	PEDF	66	male	30.0	16
**10**	PEDF	49	female	37.6	42
**11**	PEDF	83	male	24.9	36
**12**	PEDF	56	male	30.4	22
**Mean ± SD**	6 Venus	65.7 ± 9.8	7 male	37.5 ± 16.6	26.4 ± 11.3
**Min / Max**	10 PEDF	49 / 83	5 female	13.1 / 67.8	13 / 42

Human RPE cells were isolated from 12 different donors to be transfected with either the *Venus*- or the *PEDF*-encoding plasmid. The table shows with which plasmid the cells were transfected, age, gender, post-mortem time and the cultivation time before transfection. Data are expressed as mean±SD, minimum (Min) and maximum (Max) values. The results of the transfection experiments are shown in Figure 5 and 6.

Table S2 : Plasmid/mRNA amount in ng and pmol per each ratio for pSB100X/pSB- (4756 bp) or SB100X mRNA (1023 nt) and transposon plasmids pT2-CAGGS-Venus (6123 bp) and pT2-CMV-EGFP/EGFP (7259 bp).

ratio	SB100X [ng]	transposon [ng]	pSB100X/pSB-DNA [pmol]	SB100X mRNA [pmol]	pT2-CAGGS-Venus [pmol]	pT2-CMV-PEDF/EGFP [pmol]
**1:1**	250	250	0.0796	0.0760	0.0619	0.0522
**1:4**	100	400	0.0319	0.0304	0.0990	0.0835
**1:8**	55.6	444.4	0.0177	0.0169	0.1100	0.0928
**1:12**	38.5	461.5	0.0123	0.0117	0.1142	0.0963
**1:16**	29.4	470.6	0.0094	0.0089	0.1164	0.0982
**1:20**	23.8	476.2	0.0076	0.0072	0.1178	0.0994
**1:24**	20	480	0.0064	0.0061	0.1188	0.1002
**1:28**	17.2	482.8	0.0055	0.0052	0.1195	0.1008
**1:32**	15.2	484.8	0.0048	0.0046	0.1200	0.1012
**1:36**	13.5	486.5	0.0043	0.0040	0.1204	0.1015
